# Application of New Filling Material Based on Combined Heat and Power Waste for Sewage Treatment in Constructed Wetlands

**DOI:** 10.3390/ma17020389

**Published:** 2024-01-12

**Authors:** Paweł Malinowski, Wojciech Dąbrowski, Beata Karolinczak

**Affiliations:** 1 Department of Statistics and Medical Informatics, Medical University of Bialystok, 37 Szpitalna St., 15-295 Bialystok, Poland; pawel.malinowski@umb.edu.pl; 2 Faculty of Building and Environmental Sciences, Bialystok University of Technology, Wiejska St. 45E, 15-351 Białystok, Poland; 3 Faculty of Building Services Hydro and Environmental Engineering, Warsaw University of Technology, 20 Nowowiejska St., 00-653 Warsaw, Poland; beata.karolinczak@pw.edu.pl

**Keywords:** subsurface vertical constructed wetland, treatment efficiency, waste filling material, seasonality

## Abstract

The filling of constructed wetlands (CWs) affects the efficiency of sewage treatment and proper operation. Mineral aggregates are most often used as filling materials. Significant environmental burdens from mineral mining operations justify the search for waste fill. This study aimed to determine the possibility of increasing the efficiency of CW by using a Certyd aggregate as a new filling. Certyd is produced in the sintering process of coal ash, a waste from combined heat and power (CHP) plant operation. Comprehensive two-year studies were conducted using two real-scale subsurface vertical flow (SS VF) CWs supplied with domestic sewage. One bed was filled with a Certyd and the other was filled with appropriate fractions of a mineral aggregate. Both beds worked in parallel, and to compare their effectiveness, account seasonality was taken into account. The SS-VF Certyd-filled bed achieved an average efficiency of 88.0% for biological oxygen demand (BOD_5_), 80.2% for chemical oxygen demand (COD), 80.4% for suspended solids (SSs), 80.2 for ammonia nitrogen (N-NH_4_), 72.2% for total nitrogen (TN), and 55.3% for total phosphorus (TP), while the gravel-filled bed achieved 84.5%, 77.0%, 86.9%, 74.2%, 69.4%, and 57.8% for the whole research period, respectively. A higher effect of the removed unit load was achieved in the bed filled with Certyd (36.2 g BOD_5_ m^−2^ d^−1^, 50.0 g COD m^−2^ d^−1^, 5.88 g SS m^−2^ d^−1^, 7.1 g TN m^−2^ d^−1^, 7.9 g N-NH_4_ m^−2^ d^−1^, 0.79 g TP m^−2^ d^−1^) compared to the gravel-filled bed (34.7 g BOD_5_ m^−2^ d^−1^, 47.0 g COD, 6.35 g SS m^−2^ d^−1^, 6.9 g TN m^−2^ d^−1^, 7.3 g m^−2^ d^−1^ N-NH_4_, 0.83 g TP m^−2^ d^−1^).

## 1. Introduction

Constructed wetlands’ (CWs) technology has numerous advantages. It is characterized by simple construction and operation and can provide a high treatment efficiency with very low energy consumption compared to conventional systems such as activated sludge or trickling filters [1,2,3] or more advanced methods strictly connected with removal of pollutants from industrial wastewater [4].

Pollutant removal is possible, thanks to creating specific conditions that allow the plants’ growth and intensify the processes of oxidation, reduction, sorption, precipitation, sedimentation, and assimilation. Currently, subsurface vertical flow constructed wetlands (SS-VF CWs) have the most widespread usage in the treatment of domestic wastewater [5], rainwater [6], septage [7], sludge [8], and wastewater from selected industries or specific wastewaters such as leachate from landfills [9]. Currently, new applications for CWs are being sought. The use of mineral filling is associated with land degradation; therefore, it is important to look for new fillings that allow for highly efficient treatment of wastewater.

The filling of constructed wetlands affects their efficiency and proper operation. In the filling of the bed, the root system of hydrophytes grows and a biofilm is formed, which takes part in the treatment process. It also ensures a uniform and laminar flow of wastewater. The most important physical parameters of the constructed wetland bed fill include granulometric composition, porosity, and hydraulic conductivity [10].

In the 1970s, VF CWs were constructed using soil (often with a high clay content) along with organic layers [11,12]. Such fill was characterized by low hydraulic conductivity. The beds were prone to clogging and a decline in treatment efficiency was observed after a long-time operation. Therefore, materials with sufficiently high hydraulic conductivity began to be widely used [13]. The Danish standard described in [14] calls for the construction of horizontal flow CWs’ beds with mineral fill (sand and gravel). German standards [15] assume the use of sand and gravel for the construction of vertical flow and hybrid CWs. The past decade has brought research into new types of constructed wetland fill. The diversity of materials used as fill for CWs is evidenced in [16], which brings together the fills used so far: natural, modified, and waste-based. It describes and compares the characteristics, mechanism, and removal efficiency of different types of pollutants for each fill. Limitations to their use were also identified. The use of zeolite, expanded clay, and slag as fill for CWs that neutralize active substances contained in drugs is presented in [17]. In [18], using dewatered red mud (aluminum mud) to remove phosphorus from wastewater is proposed. The possibility of using construction waste (broken limestone, burnt brick fragments, and basalt grit) to fill CW beds is presented in [19]. Burnt brick fragments (waste brick) were used to build VF CW beds [20]. After three years of operation, they found a high removal effect of organics, ammonium nitrogen, and phosphorus, as well as a large growth of the biofilm.

Ceramsite, also called LECA (Light Expanded Clay Aggregate) or LWA (Lightweight Aggregate), due to its favorable physical and chemical properties, is a material used in wastewater treatment technology. LECA is an aggregate formed by sintering clay, including the addition of coal combustion ash, sewage sludge, and various chemical additives [21,22]. Depending on the fraction, the bulk density of LECA ranges from 650 to 900 kg∙m^−3^, and the specific surface area of the grains is 700–1500 m^2^∙m^−3^ [23]. These parameters allow intensive development of the biofilm and a significant increase in the contact area with wastewater. The hydraulic conductivity of expanded clay is up to 15 cm∙s^−1^ [24]. CWs filled with LECA have better hydraulic conditions and are less sensitive to clogging than gravel-filled beds. The expanded structure of the surface of LECA indirectly influences the high efficiency of nitrogen and phosphorus removal [25,26].

**Figure 1:**
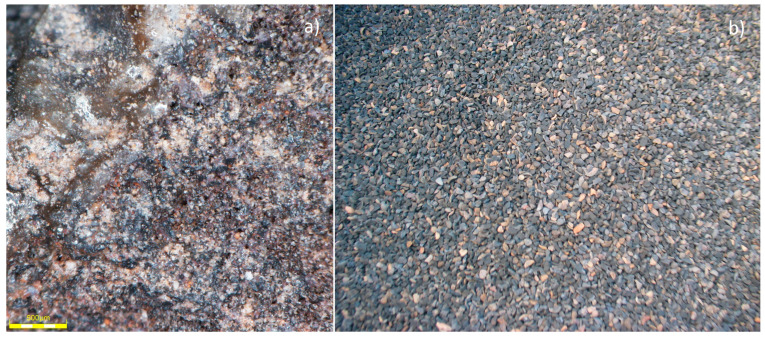
Structure of the Certyd—microscope picture (**a**), real-scale picture (**b**). Part (**a**): optical microscope, observation method: reflection, objective lens DSXPLFL3.6X, 4× zoom, 96× total magnification. Part (**b**): 1–4 mm fraction.

The Certyd aggregate used by the authors as a fill for CWs is a lightweight sintered ceramic aggregate made from coal or lignite combustion ashes (Figure 1). The production is covered by a patent [27]. The ash used for aggregate production can come from a landfill or be taken directly from the filters of the cogeneration heat and power (CHP) plant [28]. In addition, clay or bentonite was used as a binder. During the thermal treatment in the patented furnace, the ash with the binder is formed into a suitable shape (granule), dried, and sintered. Waste heat is recovered and then used to dry the granules, heat the air feeding the furnace, and heat the production hall. The final product of the sintering process is an aggregate Certyd with a bulk density ranging from 620 to 900 kg m^−3^ and a specific surface area of about 600 m^2^∙m^−3^, which is similar to the characteristic value for LECA. Aci-soluble sulfate content is 0.25%, while total sulfur content is 0.32%. The shattering resistance is no less than 6 MPa and the thermal conductivity λ = 0.14–0.16 W/mK. The Certyd characteristics and properties comply with lightweight aggregate standards (e.g., [29,30]).

The Certyd aggregate is porous, lightweight, and durable. It is resistant to fungi, mold, rodents, and insects. It is also chemically neutral, entirely capable of freezing and thawing, fire-resistant, non-degradable, suitable for reuse, and vapor-permeable. It does not lose its properties with time. Certyd can be a substitute for natural aggregates such as gravel and sand, the obtaining of which involves environmental degradation.

Certyd has so far been used mainly in the construction sector (building, road building) and horticulture. It is used as a component of lightweight structural concretes, thermal and acoustic insulation, a substrate for hydroponic cultivation, a layer in the ground, insulation of pipelines, an infiltration layer in drainage treatment plants, and construction of road embankments [31]. It has not been used for biological wastewater treatment.

The novelty of the research is the innovative usage of Certyd as an alternative filling of CWs’ beds used for wastewater treatment. The study aimed to compare the efficiency of organic matter and biogenic compound removal in gravel and Certyd-filled beds, taking into account seasonality in long-term experiments.

## 2. Materials and Methods

### 2.1. Research Installation and Sample Analysis

**Figure 2:**
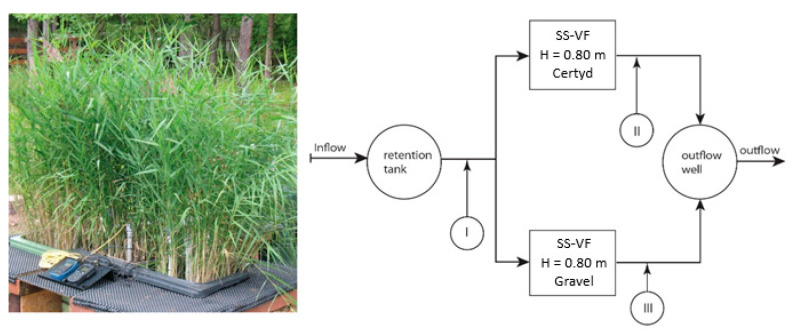
Research installation with sampling points.

**Figure 3:**
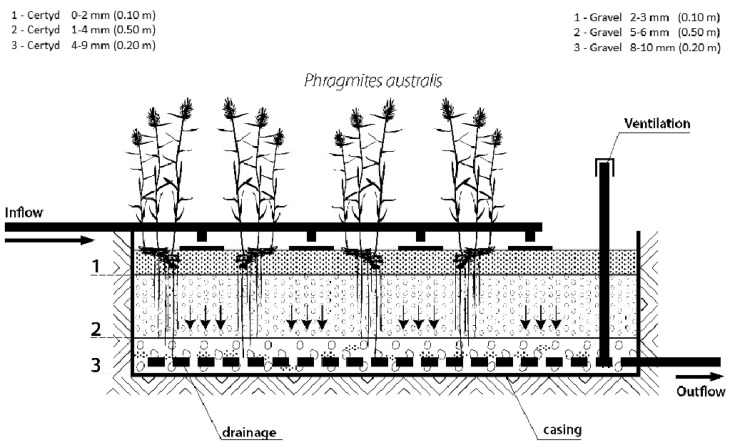
Cross-section of SS VF CW with a different filling material.

Figure 2 presents the research installation, and Figure 3 presents a cross-section of SS VF-CWs filled with Certyd and gravel used during the experiment. The filtration medium was composed of three layers with a total depth of 0.8 m. Only gravitational ventilation was applied. Sewage from a single-family house was initially treated in a two-chamber sedimentation tank and then transferred to a retention tank. Two beds were operating parallel with the same hydraulic load of 0.1 m d^−1^. The loads used are typical for domestic wastewater treatment systems [10]. The beds were planted with reeds (*Phragmites australis*). The research was carried out over two years, and 45 series were collected. Each series consisted of a raw sewage sample (sampling point I) and two samples of treated sewage (sampling points II and III). Vegetation (April until November) and non-vegetation seasons were distinguished. The quality and quantity of raw sewage is an important parameter in the design and assessment of the effectiveness of treatment systems. The daily wastewater flow from the household was 0.25 $m^{3}\cdot d^{-1}$. The content of organic matter (BOD_5_, COD, TOC), suspended solids (SSs), total nitrogen (TN), ammonia nitrogen (NH_4_-N), and total phosphorus (TP) were analyzed. The tests recommended by Merck were performed in the Department of Environmental Engineering and Natural Sciences laboratory at Bialystok University of Technology. Wastewater testing was conducted following the requirements of the American Public Health Association [32]. Spectrophotometer Spectroquant Pharo 100 (Merck Millipore, Burlington, MA, USA) was used. BOD_5_ was determined using OXI-TOP^®^ (Xylem Analytics, Washington, DC, USA). In addition, measurements were taken of pH, conductivity, and dissolved oxygen concentration, using the multi-parameter device WTW MutiLine P4 (Labexchange, Burladingen, Germany).

In addition, the study determined parameters such as the permeability coefficient for Certyd and gravel, which are the main fill layers of the CW beds used in this research. These tests have not been carried out before because Certyd has not been used as a filter material for water and wastewater treatment.

The permeability coefficient of Certyd and gravel was determined using the constant head permeability tests. The coefficient is calculated in this method from Darcy’s law after measuring the volume of water flowing through the sample in a specific amount of time while the hydraulic gradient is constant during the test. A Polish version of an apparatus called the Wiłun’s cell for the permeability test was used for the tests. This apparatus is on the equipment of the geotechnical laboratory of the Department of Geotechnics, Roads and Geodesy, Bialystok University of Technology.

### 2.2. Statistical Methodology and Data Refining

In the section Results and Discussion, statistical parameters characterizing the distribution of the measured parameters are presented in the following pattern:

(1) $$\begin{array}{c} \frac{mean\pm sd\left(med\pm mad\right)}{min,q1,q3,max}\\ p_{RW},p_{SW} \end{array}$$

where mean is the arithmetical mean; sd is the standard deviation; med is the median; mad is the scaled median deviation from the median, the so-called MAD (median absolute deviation) measure; min and max are the extreme values; q1 and q3 are the 1st and 3rd quartile; $p_{RW}$ and $p_{SW}$ are the *p*-values for tests Rothman–Woodroofe (symmetry of distribution) and Shapiro–Wilk (normality).

The interpretation of the above notation may suggest a certain symmetry concerning the mean or median. Therefore, a symmetry test of the distribution with Rothman–Woodroofe statistics was performed to validate this interpretation [33].

The mean value has the correct interpretation when the selected distribution is symmetrical. The standard deviation has a proper interpretation only when the distribution of the measured quantity is normal. The normal distribution is symmetrical. Therefore, the Shapiro–Wilk normality test was performed [34], but only when the symmetry test gave a statistically insignificant result. Irrespective of the test results, all aggregate statistical parameters are included, due to a similar approach used in the literature. Appropriate *p*-values of the tests allow the selection of the most appropriate ones.

The following value was used as a scale factor for MAD:

(2) $$k_{MAD}=\frac{1}{cdf_{N\left(0,1\right)}^{-1}\left(0.75\right)}$$

where $k_{MAD}$ is the scale factor; $cdf_{N\left(0,1\right)}^{-1}\left(\cdot \right)$ is an inverse of the cumulative standard normal distribution function.

For normally distributed data, the selected scale factor makes MAD measure asymptotically approximate standard deviation. Quartiles and MAD are non-parametric and their interpretation is appropriate for any distribution.

Loads *L* (per unit surface) were calculated as a product of hydraulic load (*q*) and concentration (*C*):

(3) L=Cq

Treatment efficiencies (η) were calculated according to the terminology given in [10]:

(4) $$\eta =1-\frac{C_{effluent}}{C_{influent}}$$

To compare the efficiency of CWs’ bed working under the same conditions, paired versions of statistical tests were performed: either the *t*-test or the Wilcoxon signed rank test, depending on the test of normality of the differences. For differences with insignificant Shapiro–Wilk-test *p*-values, the paired *t*-test was performed. The Wilcoxon signed rank test was performed otherwise. Along with tests’ statistics and *p*-values, means or medians of differences were provided, depending on their normality.

Distributions of selected variables were presented graphically. Due to the finite number of measurements, the most appropriate graphical representation is a histogram and box–whisker plot. For a single-variable histogram, the number of histogram bars was determined using the Sturges algorithm [35]. For multiple variables, the following steps were performed in order:

- for each of the variables, the number of bars was determined;
- for each of the variables, bar widths were determined in proportion to the range;
- the smallest width was selected and the global number of bars for the full range was recalculated;
- the target width of the bars ($d_{glob}$) was selected using the ‘pretty’ algorithm, where the previously calculated global number of bars was provided as a hint.

The histogram approximates the true density function of the represented variable. Another possible approximation is the graph of the curve resulting from the ‘density’ algorithm. This algorithm approximates the true density function by convoluting the original data with a certain window function using the Fourier transform, from which the values of the density function approximation are computed [36]. The calculated histogram and the curve have a similar shape but on a different scale. The natural interpretation of the histogram is frequency, which is responsible for the height of each bar. This interpretation was left unchanged, but the density curve was rescaled. Its values have been scaled using a factor:

(5) $$k_{density}=n\cdot d_{glob}$$

where $k_{density}$ is the scale factor; *n* is the total number of samples.

The box–whisker plots show the distribution of the selected variable. The box represents the median (horizontal box line) and the first and third quartiles (box edges). The whiskers extend at most 1.5 times the difference between the first and third quartiles but do not extend beyond the extreme values. All observations outside the whiskers are marked as points.

All previously mentioned algorithms are implemented in the R statistical environment [37]. The ‘symmetry_test’ function of the symmetry package [38] implements the symmetry test. The number of bootstrap repetitions was specified as 10,000. This was a reasonable compromise between the stability of the test results and the time of operation. The ‘shapiro_test’ function of the stats package implements the Shapiro–Wilk normality test. The package stats also contains implementations of the Wilcoxon signed rank test (‘wilcox.test’) and paired *t*-test (‘t.test’).

## 3. Results and Discussion

The permeability test performed in the geochemical laboratory showed a value of 8.97 × 10^−1^ cm s^−1^ for Certyd (fraction: 1–4 mm) and 6.31 × 10^−1^ cm s^−1^ for gravel (fraction: 5–6 mm). Both materials had a high permeability coefficient. It is well known that a high value of the permeability coefficient results in better aeration and prevents clogging of CWs. Table 1 presents the characteristics of the wastewater quality used in the study. To be able to evaluate the efficiency of the treatment process, wastewater parameters are divided into vegetation and non-vegetation periods.

The value of BOD_5_ in the wastewater supplying the beds varied from 370 to 490 ${\mathrm{gO}}_{2}\cdot m^{-3}$ (410 ${\mathrm{gO}}_{2}\cdot m^{-3}$ on average), while COD is from 700 to 843 ${\mathrm{gO}}_{2}\cdot m^{-3}$ (average: 738 ${\mathrm{gO}}_{2}\cdot m^{-3}$). Similar parameters after primary sedimentation (BOD_5_: 488.5 ${\mathrm{gO}}_{2}\cdot m^{-3}$, COD: 880.0 ${\mathrm{gO}}_{2}\cdot m^{-3})$ were obtained in [39], while analyzing the effectiveness of five hybrid systems treating domestic wastewater plants. In the case of nutrients, the composition of wastewater was also similar. Long-term studies (1997–2010), conducted as part of the monitoring of domestic wastewater treatment plants with pre-treatment and CWs with horizontal flow [1], showed the following parameters: BOD_5_ from 62 to 301 ${\mathrm{gO}}_{2}\cdot m^{-3}$ (average: 163.2 ${\mathrm{gO}}_{2}\cdot m^{-3}$), COD from 101 to 580 ${\mathrm{gO}}_{2}\cdot m^{-3}$ (average: 329.8 ${\mathrm{gO}}_{2}\cdot m^{-3}$), TN from 37.1 to 137 $g\cdot m^{-3}$ (average: 71.5 $g\cdot m^{-3}$), TP from 5.2 to 42.8 $g\cdot m^{-3}$ (average: 24.8 $g\cdot m^{-3}$).

Significant differences are observed in the unit volume of domestic wastewater treated on-site, which varies from 0.039 to 0.539 $m^{3}\cdot M^{-1}\cdot d^{-1}$ [40]. The typical composition of domestic wastewater is difficult to determine. The main factor influencing the pollutant concentrations in domestic sewage is water consumption; saving water increases the concentration of sewage parameters. The results of the study (Table 1) indicate a high organic matter content, resulting from low unit water consumption. The CWs’ beds were fed with sewage pre-treated in a two-chamber tank (Figure 2), which provides a reduction in SS, BOD_5_, and COD concentration. The capacity of the tank, the number of chambers, and the possible use of chemical precipitation can significantly alter the parameters of CWs’ bed influent.

**Figure 4:**
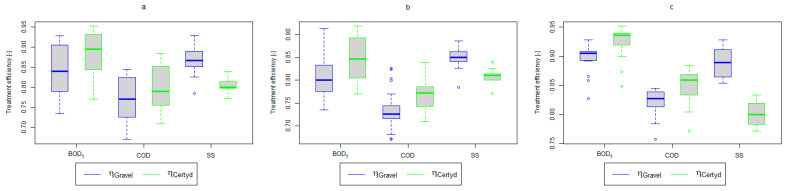
Treatment efficiency—BOD_5_, COD, and SS: (**a**) whole study period, (**b**) non-vegetative period, (**c**) vegetation period.

**Figure 5:**
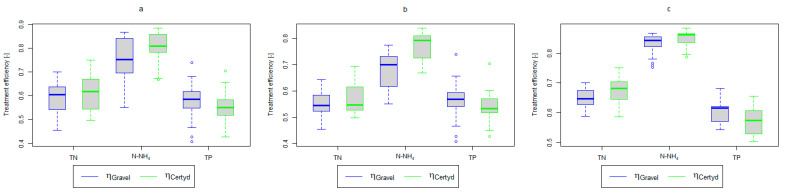
Treatment efficiency—TN, N-NH_4_, and TP: (**a**) whole study period, (**b**) non-vegetative period, (**c**) vegetation period.

Table 2 and Table 3 show the pollutant loads in wastewater supplying the CWs’ beds and the loads removed in relation to the unit area of the bed. Figure 4 and Figure 5 present treatment efficiency.

**Figure 6:**
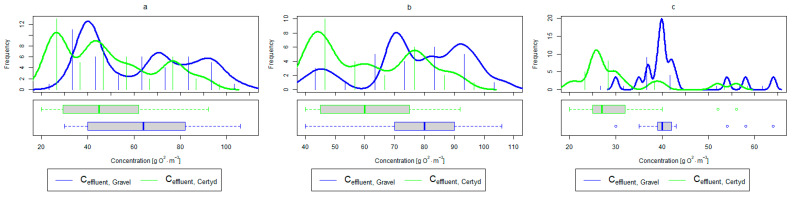
Parameters of treated sewage (gravel and Certyd)—BOD_5_: (**a**) whole study period, (**b**) non-vegetative period, (**c**) vegetation period.

**Figure 7:**
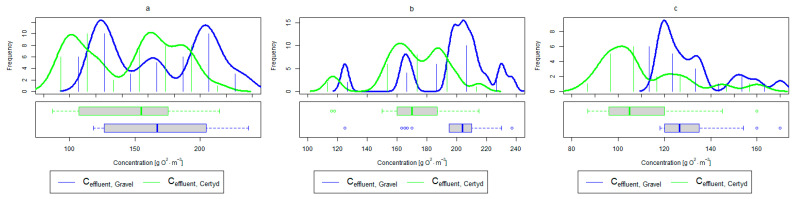
Parameters of treated sewage (gravel and Certyd)—COD: (**a**) whole study period, (**b**) non-vegetative period, (**c**) vegetation period.

**Figure 8:**
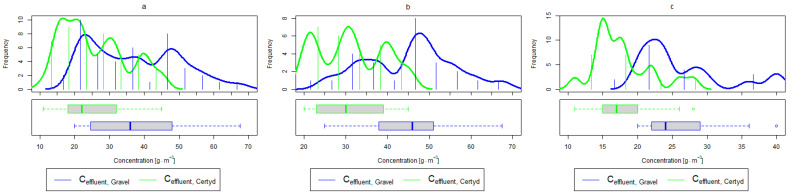
Parameters of treated sewage (gravel and Certyd)—TOC: (**a**) whole study period, (**b**) non-vegetative period, (**c**) vegetation period.

**Figure 9:**
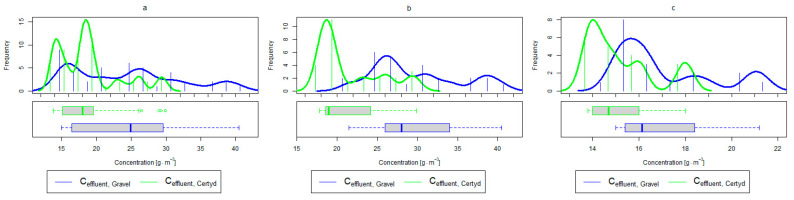
Parameters of treated sewage (gravel and Certyd)—N-NH_4_: (**a**) whole study period, (**b**) non-vegetative period, (**c**) vegetation period.

**Figure 10:**
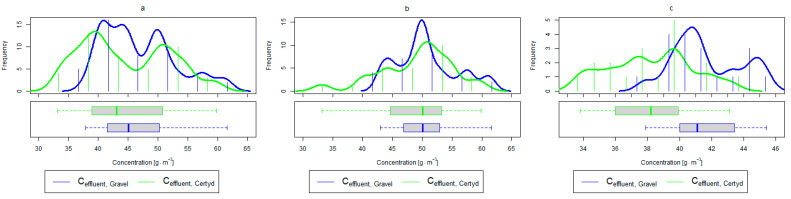
Parameters of treated sewage (gravel and Certyd)—TN: (**a**) whole study period, (**b**) non-vegetative period, (**c**) vegetation period.

**Figure 11:**
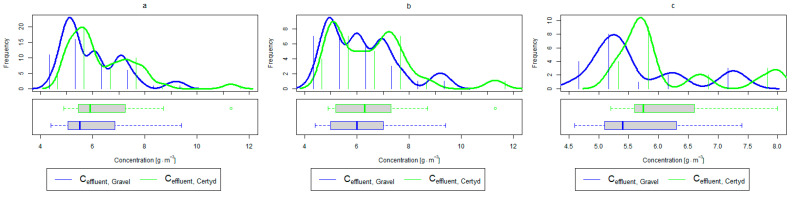
Parameters of treated sewage (gravel and Certyd)—TP: (**a**) whole study period, (**b**) non-vegetative period, (**c**) vegetation period.

**Figure 12:**
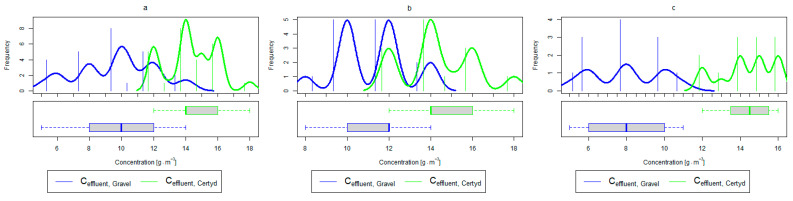
Parameters of treated sewage (gravel and Certyd)—SS: (**a**) whole study period, (**b**) non-vegetative period, (**c**) vegetation period.

For organic compounds, the beds’ load averaged at BOD_5_: 41.0 ${\mathrm{gO}}_{2}\cdot m^{-2}\cdot d^{-1}$ (37.0 to 49.0 ${\mathrm{gO}}_{2}\cdot m^{-2}\cdot d^{-1}$), COD: 73.8 ${\mathrm{gO}}_{2}\cdot m^{-2}\cdot d^{-1},$ SS: 7.31 $g\cdot m^{-2}\cdot d^{-1}$ (Table 2). No significant differences were observed in the beds’ loading during the study period. Figure 6, Figure 7, Figure 8, Figure 9, Figure 10, Figure 11 and Figure 12 present the parameters of treated sewage.

Table 4 presents the test statistics along with *p*-values of normality and appropriate paired tests for treatment efficiencies, η.

Results for the CW bed filled with gravel were taken as a reference for mean or median difference Δη: reported values greater than 0 (with significant *p*-value) mean that the CW bed filled with Certyd performed better. Almost all reported differences were highly statistically significant—only two *p*-values were greater than 0.01 (out of three greater than 0.001). Only removal efficiencies of TN in the non-vegetation period for both CW beds yield a marginally significant difference with a *p*-value approaching the α=0.05 threshold.

A higher organic matter removal effect was found in the Certyd bed throughout the study period. The removal effect of the organic pollutant load expressed as BOD_5_ was 88.0% with an average value at the outflow of 48.0 ${\mathrm{gO}}_{2}\cdot m^{-3}$ for the Certyd-filled bed and 84.5% and 63.0 ${\mathrm{gO}}_{2}\cdot m^{-3}$ for the gravel-filled bed (Figure 6). Similarly, for COD, the efficiencies were 80.2 and 77.0% with values at the outflow of 146 and 169 ${\mathrm{gO}}_{2}\cdot m^{-3}$ (Figure 7). A similar trend was observed for TOC (Figure 8). Based on studies of a number of wastewater treatment plants in various countries, [9] reports a 90% BOD_5_ removal efficiency in SS VF CWs and 85–87.5% COD removal efficiency in a hybrid treatment plant in Wiedersberg, during the growing season [41]. Organic matter removal efficiencies measured using COD at treatment plants in France ranged from 90.0 to 91.3% according to [42]. Removal efficiencies for BOD_5_ and COD at treatment plants located in Beijing were 87.0 and 82.0% [43].

The study showed a high organic matter removal effect per unit area of the CW bed. Concerning BOD_5_, the load removed (average value) was, for the gravel-filled bed, 34.7 ${\mathrm{gO}}_{2}\cdot m^{-2}d^{-1}$ and 36.2 ${\mathrm{gO}}_{2}\cdot m^{-2}d^{-1}$ for the Certyd-filled bed. In the case of COD, it was, respectively, 56.9 ${\mathrm{gO}}_{2}\cdot m^{-2}d^{-1}$ and 59.2 ${\mathrm{gO}}_{2}\cdot m^{-2}d^{-1}$. The literature notes even better results. The unit load removed in CWs treating fruit and vegetable processing wastewater was 71.24 ${\mathrm{gO}}_{2}\cdot m^{-2}d^{-1}$ for BOD_5_ and as high as 221.44 ${\mathrm{gO}}_{2}\cdot m^{-2}d^{-1}$ for COD [44].

Factors influencing the efficiency of CWs include hydraulic load, pollutant load, seasonality, the configuration of the beds, plant species, and bed oxygenation [41,45].

The high removal efficiency of organic matter in vertical flow beds is explained by their good oxygenation, which affects biochemical transformations. Better oxygenation of the Certyd-filled bed resulted in higher organic matter removal efficiency.

An important factor influencing the efficiency and proper operation of CWs’ beds is the appropriate pre-treatment of wastewater. The use of a primary settling tank to remove SS protects CWs from colmatation. Removal efficiencies for remaining SS in CWs ranging from 73.0 to 90.0% were reported in [40]. Similar results were achieved in the presented study (from 86.9% for the gravel-filled bed to 80.4% for the Certyd-filled bed) (Figure 12). The slightly lower efficiency for the Certyd-filled bed may have been due to the higher filtration velocity through this bed. The removal effect of SS was similar during the growing and non-growing seasons.

Analyzing the efficiency of N-NH_4_ removal, it was found for the entire study period that the efficiency for the gravel-filled bed was 74.2 and 80.2% for Certyd. For TN, it was 69.4 and 75.2%, respectively (Figure 5). This is confirmed by the parameters of the treated wastewater shown in Figure 9 and Figure 10. A range of TN removal from 23.0 to 43.0% was reported in [40]. The concentration of N-NH_4_ was significantly lower for sewage treated in a Certyd-filled bed. A better nitrification effect was observed in the Certyd-filled bed: the concentration of nitrate nitrogen in the outflow from the Certyd-filled bed was 15.7 $g\cdot m^{-3}$, and from the gravel-filled bed, it was 11.4 $g\cdot m^{-3}$. Proper oxygenation of CW beds is indicated as the main factor influencing the nitrification process occurring in VF-CWs [10]. According to [9], the efficiency of TN removal in processes in SS VF beds was up to 43.0%, while N-NH_4_ was up to 73%. N-NH_4_ removal efficiencies ranging from 94.8 to 98.5%, and those of TN from 52.9 to 78.4%, were reported in [46].

In the presented study, a high removal effect of TN and N-NH_4_ was achieved per unit area of CWs (Table 3). TN removal was 6.9 $g\cdot m^{-2}d^{-1}$ for the gravel-filled bed, while it was 7.1 $g\cdot m^{-2}d^{-1}$ for the Certyd-filled bed. In the case of N-NH_4_, it was 7.3 $g\cdot m^{-2}d^{-1}$ for the gravel-filled bed, and 7.9 $g\cdot m^{-2}d^{-1}$ for the Certyd-filled bed. The Certyd-filled bed had better oxygenation, and hence higher N-NH_4_ removal efficiency and lower nitrate removal efficiency. The efficiency of TN removal in the CWs is dependent on the efficiency of the denitrification process, which requires anoxic conditions. Such conditions can occur in horizontal flow or a hybrid CW system [10,13].

As in the case of nitrogen, an analysis of the literature shows wide discrepancies in the removal efficiency of TP. Season and temperature have virtually no effect on removal efficiency of SS and TP, as their removal is related more to physical than biological processes. In the presented study, TP removal efficiency was 57.8% for the gravel-filled bed, and its average concentration in the treated wastewater was 5.98 $g\cdot m^{-3}$ (from 4.40 to 9.40 $g\cdot m^{-3}$). In the Certyd-filled bed, the removal efficiency of TP was slightly lower at 55.3% (Figure 5), and its average concentration in the treated wastewater was 6.4 $g\cdot m^{-3}$ (from 4.9 to 11.3$\;g\cdot m^{-3}$) (Figure 11). A TP removal efficiency of 25.0% was obtained and reported in [14]. As in the case of TN and ammonia nitrogen, a high phosphorus removal efficiency was achieved in their study concerning the unit area of the bed. For the gravel fill, it was an average of 0.83 $g\cdot m^{-2}d^{-1}$, and for the Certyd fill, it was 0.79 $g\cdot m^{-2}d^{-1}$ (Table 3). An efficiency of 0.19 $g\cdot m^{-2}d^{-1}$ was reported in [9]. Phosphorus contained in wastewater can be sedimented and deposited or chemically bound as complex compounds of aluminum, iron, calcium, or magnesium [47]. Regardless of these processes, phosphorus removal efficiency is low in most CWs. If bed conditions are changed, chemically bound phosphorus can be released back into the treated effluent. Removal efficiency can be significantly improved by using special phosphorus-binding additives [48]. To assess phosphorus removal efficiency, multi-year studies should be conducted. An increase in phosphorus and nitrogen removal efficiency can be achieved by removing biomass from the CWs during the non-vegetation period [40].

Table 5 shows the indicators characterizing the biodegradation susceptibility of sewage, i.e., BOD_5_:COD and BOD_5_:TN ratios. Table 6 shows dissolved oxygen concentration, pH, conductivity, and alkalinity.

The average BOD_5_:COD ratio in raw sewage was 0.555, while BOD_5_:TN was 3.57. In treated sewage, it was 0.361 and 1.31 for the gravel-filled bed and 0.320 and 1.07 for the Certyd-filled bed. The obtained results seem to confirm the possibility of effective biological wastewater treatment. A similar favorable value of indicators for domestic sewage treated with constructed wetlands was obtained in [40]. Those indicators are also important in assessing the possibility of intensifying the treatment with a hybrid system [49]. The effectiveness of nitrification taking place in both CWs’ beds is confirmed by the analysis of alkalinity in raw and treated wastewater, as well as the concentrations of dissolved oxygen (Table 6). The mean alkalinity decreased from 13.6 to 7.4 $\mathrm{mval}\cdot {\mathrm{dm}}^{-3}$ in the Certyd-filled bed and 6.1 $\mathrm{mval}\cdot {\mathrm{dm}}^{-3}$ in the gravel-filled bed.

The Certyd-filled bed had a better ability to oxygenate wastewater. The concentration of dissolved oxygen in raw sewage was on average 0.47 $g\cdot m^{-3}$ (varied from 0.19 to 1.20 $g\cdot m^{-3}$), while in sewage treated in the Certyd-filled bed, it was 2.43 $g\cdot m^{-3}$ (1.62 to 3.60 $g\cdot m^{-3}$), and in the gravel-filled bed, it was 1.91 $g\cdot m^{-3}$ (0.96 to 2.90 $g\cdot m^{-3}$). The pH value in wastewater from both beds was similar, and in the case of conductivity, no significant differences were found between wastewater treated in both beds.

## 4. Conclusions

The novelty of the research is due to the usage of Certyd as an alternative filling of CWs’ beds, which reduces the environmental and landscape degradation associated with the exploitation of gravel mines.

The results of the study confirmed the feasibility of Certyd aggregate usage as a fill of constructed wetlands for high-efficiency wastewater treatment. The Certyd-filled bed had a higher removal effect of organic matter and nitrogen compounds compared to the gravel-filled bed. The average efficiency of organic matter removal was 88.0% (BOD_5_) and 80.2% (COD) in the Certyd-filled bed, while in the gravel-filled bed, it was 84.5% and 77.0, respectively. The removal effect of SS and TP was lower (80.4% and 55.3%) compared to the gravel-filled bed (86.9% and 57.8%). The average removal efficiency of nitrogen compounds throughout the study period in the beds filled with Certyd and gravel was 80.2 and 74.2% (N-NH_4_), and 61.1% and 59.1% (TN). Differences in reported efficiencies, in both the vegetative and non-vegetative periods, were statistically significant, most of them with very low *p*-values. The nitrification process occurred more efficiently in the Certyd-filled bed. This was related to the higher concentration of dissolved oxygen in the treated wastewater in the Certyd-filled bed, which was 2.43 g m^−3^, and 1.91 g m^−3^ in the gravel-filled bed.

## Figures and Tables

**Table 1:** Parameters of raw sewage used in the experiment.

Parameter	Whole Research Period	Vegetation Period	Non-Vegetation Period
$\begin{array}{c} BOD_{5}\\ \left[gO_{2}\cdot m^{-3}\right] \end{array}$	$\begin{array}{c} \frac{410\pm 25\;\left(400\pm 30\right)}{370,395,430,490}\\ p1=0.07,\;p2=0.04 \end{array}$	$\begin{array}{c} \frac{415\pm 21\;\left(420\pm 19\right)}{370,400,430,440}\\ p1=0.81,\;p2=0.03 \end{array}$	$\begin{array}{c} \frac{406\pm 28\;\left(400\pm 15\right)}{370,390,410,490}\\ p1<0.01 \end{array}$
$\begin{array}{c} COD\\ \left[gO_{2}\cdot m^{-3}\right] \end{array}$	$\begin{array}{c} \frac{738\pm 31\;\left(730\pm 30\right)}{700,720,755,843}\\ p1=0.16,\;p2<0.01 \end{array}$	$\begin{array}{c} \frac{736\pm 19\;\left(730\pm 22\right)}{700,723,750,760}\\ p1=0.64,\;p2=0.08 \end{array}$	$\begin{array}{c} \frac{740\pm 38\;\left(730\pm 30\right)}{700,720,760,843}\\ p1=0.10;\;p2<0.01 \end{array}$
$\begin{array}{c} SS\\ \left[g\cdot m^{-3}\right] \end{array}$	$\begin{array}{c} \frac{73.1\pm 5.2\;\left(72.0\pm 4.4\right)}{65.0,70.0,75.0,90.0}\\ p1=0.03 \end{array}$	$\begin{array}{c} \frac{71.9\pm 3.9\;\left(71.5\pm 3.7\right)}{65.0,70.0,75.0,80.0}\\ p1=0.71,\;p2=0.76 \end{array}$	$\begin{array}{c} \frac{73.8\pm 5.9\;\left(73.0\pm 4.4\right)}{65.0,70.0,75.0,90.0}\\ p1=0.10,\;p2=0.03 \end{array}$
$\begin{array}{c} TN\\ \left[g\cdot m^{-3}\right] \end{array}$	$\begin{array}{c} \frac{116\pm 13\;\left(113\pm 12\right)}{97,106,123,141}\\ p1<0.01 \end{array}$	$\begin{array}{c} \frac{118\pm 13\;\left(118\pm 11\right)}{98,111,126,140}\\ p1=0.90,\;p2=0.50 \end{array}$	$\begin{array}{c} \frac{113.7\pm 12.7\;\left(109.7\pm 8.5\right)}{97.0,105.2,118.3,141.5}\\ p1<0.01 \end{array}$
$\begin{array}{c} N-NH_{4}\\ \left[g\cdot m^{-3}\right] \end{array}$	$\begin{array}{c} \frac{97\pm 11\;\left(94\pm 12\right)}{85,89,105,120}\\ p1=0.05 \end{array}$	$\begin{array}{c} \frac{103\pm 12\;\left(104\pm 18\right)}{85,90,110,120}\\ p1=0.06,\;p2=0.06 \end{array}$	$\begin{array}{c} \frac{93.7\pm 8.1\;\left(90.0\pm 5.8\right)}{85.0,88.3,96.5,116.5}\\ p1<0.01 \end{array}$
$\begin{array}{c} TP\\ \left[g\cdot m^{-3}\right] \end{array}$	$\begin{array}{c} \frac{14.3\pm 2.3\;\left(14.0\pm 2.7\right)}{10.3,12.8,16.1,20.5}\\ p1=0.31,\;p2=0.35 \end{array}$	$\begin{array}{c} \frac{14.3\pm 1.4\;\left(14.0\pm 1.4\right)}{12.1,13.2,15.6,16.4}\\ p1=0.25,\;p2=0.16 \end{array}$	$\begin{array}{c} \frac{14.2\pm 2.8\;\left(14.1\pm 4.2\right)}{10.3,12.0,16.9,20.5}\\ p1=0.64,\;p2=0.16 \end{array}$

**Table 2:** Raw sewage and removed loads (BOD_5_, COD, and SS).

Period	Load, g·m^−2^·d^−1^	BOD_5_	COD	SS
Whole period	$L_{in}$	$\begin{array}{c} \frac{41.0\pm 2.5\;\left(40.0\pm 3.0\right)}{37.0,39.5,43.0,49.0}\\ p1=0.07,\;p2=0.04 \end{array}$	$\begin{array}{c} \frac{73.8\pm 3.1\;\left(73.0\pm 3.0\right)}{70.0,72.0,75.5,84.3}\\ p1=0.16,\;p2<0.01 \end{array}$	$\begin{array}{c} \frac{7.31\pm 0.52\;\left(7.20\pm 0.44\right)}{6.50,7.00,7.50,9.00}\\ p1=0.03 \end{array}$
	$L_{rem,\;Gravel}$	$\begin{array}{c} \frac{34.7\pm 4.2\;\left(35.2\pm 5.6\right)}{27.5,31.0,38.3,44.4}\\ p1=0.78,\;p2=0.13 \end{array}$	$\begin{array}{c} \frac{56.9\pm 5.2\;\left(56.6\pm 5.3\right)}{47.0,53.0,60.8,67.8}\\ p1=0.97,\;p2=0.71 \end{array}$	$\begin{array}{c} \frac{6.35\pm 0.51\;\left(6.40\pm 0.44\right)}{5.10,6.05,6.55,7.60}\\ p1=0.49,\;p2=0.48 \end{array}$
	$L_{rem,Certyd}$	$\begin{array}{c} \frac{36.2\pm 4.0\;\left(36.2\pm 5.8\right)}{29.2,32.4,39.8,45.0}\\ p1=0.92,\;p2=0.08 \end{array}$	$\begin{array}{c} \frac{59.2\pm 5.0\;\left(59.5\pm 5.9\right)}{50.0,55.0,62.8,68.5}\\ p1=0.45,\;p2=0.28 \end{array}$	$\begin{array}{c} \frac{5.88\pm 0.49\;\left(5.80\pm 0.44\right)}{5.20,5.45,6.10,7.20}\\ p1=0.20,\;p2=0.13 \end{array}$
Veg. period	$L_{in}$	$\begin{array}{c} \frac{41.5\pm 2.1\;\left(42.0\pm 1.9\right)}{37.0,40.0,43.0,44.0}\\ p1=0.81,\;p2=0.03 \end{array}$	$\begin{array}{c} \frac{73.6\pm 1.9\;\left(73.0\pm 2.2\right)}{70.0,72.3,75.0,76.0}\\ p1=0.64,\;p2=0.08 \end{array}$	$\begin{array}{c} \frac{7.19\pm 0.39\;\left(7.15\pm 0.37\right)}{6.50,7.00,7.50,8.00}\\ p1=0.71,\;p2=0.76 \end{array}$
	$L_{rem,\;Gravel}$	$\begin{array}{c} \frac{37.3\pm 2.6\;\left(38.3\pm 2.4\right)}{30.6,35.8,39.1,40.0}\\ p1=0.30,\;p2=0.02 \end{array}$	$\begin{array}{c} \frac{60.3\pm 3.1\;\left(60.8\pm 3.3\right)}{53.0,58.6,62.9,64.2}\\ p1=0.22,\;p2=0.21 \end{array}$	$\begin{array}{c} \frac{6.39\pm 0.35\;\left(6.40\pm 0.30\right)}{5.90,6.18,6.53,7.20}\\ p1=0.71,\;p2=0.56 \end{array}$
	$L_{rem,Certyd}$	$\begin{array}{c} \frac{38.4\pm 2.8\;\left(39.5\pm 2.3\right)}{31.4,36.5,40.4,41.3}\\ p1=0.15,\;p2=0.02 \end{array}$	$\begin{array}{c} \frac{62.5\pm 3.4\;\left(62.8\pm 3.9\right)}{54.0,60.6,65.3,66.4}\\ p1=0.15,\;p2=0.12 \end{array}$	$\begin{array}{c} \frac{5.76\pm 0.40\;\left(5.75\pm 0.44\right)}{5.20,5.40,6.00,6.60}\\ p1=0.16,\;p2=0.41 \end{array}$
Non-veg. period	$L_{in}$	$\begin{array}{c} \frac{40.6\pm 2.8\;\left(40.0\pm 1.5\right)}{37.0,39.0,41.0,49.0}\\ p1<0.01 \end{array}$	$\begin{array}{c} \frac{74.0\pm 3.8\;\left(73.0\pm 3.0\right)}{70.0,72.0,76.0,84.3}\\ p1=0.10,\;p2<0.01 \end{array}$	$\begin{array}{c} \frac{7.38\pm 0.59\;\left(7.30\pm 0.44\right)}{6.50,7.00,7.50,9.00}\\ p1=0.10,\;p2=0.03 \end{array}$
	$L_{rem,\;Gravel}$	$\begin{array}{c} \frac{32.9\pm 4.1\;\left(31.0\pm 2.8\right)}{27.5,29.8,35.4,44.4}\\ p1=0.24,\;p2<0.01 \end{array}$	$\begin{array}{c} \frac{54.4\pm 5.0\;\left(53.5\pm 4.4\right)}{47.0,52.2,56.5,67.8}\\ p1=0.21,\;p2=0.02 \end{array}$	$\begin{array}{c} \frac{6.31\pm 0.65\;\left(6.20\pm 0.44\right)}{5.10,6.00,6.65,7.60}\\ p1=1.00,\;p2=0.92 \end{array}$
	$L_{rem,Certyd}$	$\begin{array}{c} \frac{34.5\pm 3.9\;\left(33.0\pm 3.7\right)}{29.2,31.5,36.3,45.0}\\ p1=0.07,\;p2=0.06 \end{array}$	$\begin{array}{c} \frac{56.8\pm 4.8\;\left(55.6\pm 4.6\right)}{50.0,53.5,59.5,68.5}\\ p1=0.37,\;p2=0.05 \end{array}$	$\begin{array}{c} \frac{6.01\pm 0.57\;\left(6.00\pm 0.59\right)}{5.30,5.60,6.35,7.20}\\ p1=0.59,\;p2=0.53 \end{array}$

**Table 3:** Raw sewage and removed loads (TN, N-NH_4_, and TP).

Period	Load, g·m^−2^·d^−1^	TN	N-NH_4_	TP
Whole period	$L_{in}$	$\begin{array}{c} \frac{11.6\pm 1.3\;\left(11.3\pm 1.2\right)}{9.7,10.6,12.3,14.1}\\ p1<0.01 \end{array}$	$\begin{array}{c} \frac{9.7\pm 1.1\;\left(9.4\pm 1.2\right)}{8.5,8.9,10.5,12.0}\\ p1<0.05 \end{array}$	$\begin{array}{c} \frac{1.43\pm 0.23\;\left(1.40\pm 0.27\right)}{1.03,1.28,1.61,2.05}\\ p1=0.31.\;p2=0.35 \end{array}$
	$L_{rem,\;Gravel}$	$\begin{array}{c} \frac{6.9\pm 1.4\;\left(6.9\pm 1.5\right)}{5.0,5.8,7.8,9.6}\\ p1=0.11,\;p2=0.02 \end{array}$	$\begin{array}{c} \frac{7.3\pm 1.6\;\left(6.9\pm 2.1\right)}{4.8,6.3,8.8,10.4}\\ p1=0.02 \end{array}$	$\begin{array}{c} \frac{0.83\pm 0.17\;\left(0.82\pm 0.15\right)}{0.44,0.75,0.95,1.25}\\ p1=0.51.\;p2=0.85 \end{array}$
	$L_{rem,Certyd}$	$\begin{array}{c} \frac{7.1\pm 1.4\;\left(6.6\pm 1.4\right)}{5.3,5.8,8.3,10.5}\\ p1=0.17,\;p2=0.01 \end{array}$	$\begin{array}{c} \frac{7.9\pm 1.4\;\left(7.6\pm 1.7\right)}{6.0,6.7,9.0,10.6}\\ p1=0.26,\;p2=0.02 \end{array}$	$\begin{array}{c} \frac{0.79\pm 0.15\;\left(0.77\pm 0.16\right)}{0.54,0.69,0.89,1.19}\\ p1=0.53.\;p2=0.52 \end{array}$
Veg. period	$L_{in}$	$\begin{array}{c} \frac{11.8\pm 1.3\;\left(11.8\pm 1.1\right)}{9.8,11.1,12.6,14.0}\\ p1=0.90,\;p2=0.50 \end{array}$	$\begin{array}{c} \frac{10.3\pm 1.2\;\left(10.4\pm 1.8\right)}{8.5,9.0,11.0,12.0}\\ p1=0.06,\;p2=0.06 \end{array}$	$\begin{array}{c} \frac{1.43\pm 0.14\;\left(1.40\pm 0.14\right)}{1.21,1.32,1.56,1.64}\\ p1=0.25,\;p2=0.16 \end{array}$
	$L_{rem,\;Gravel}$	$\begin{array}{c} \frac{7.7\pm 1.2\;\left(7.7\pm 1.2\right)}{5.9,6.9,8.4,9.6}\\ p1=0.61,\;p2=0.27 \end{array}$	$\begin{array}{c} \frac{8.6\pm 1.3\;\left(8.8\pm 1.5\right)}{6.4,7.1,9.5,10.4}\\ p1=0.02 \end{array}$	$\begin{array}{c} \frac{0.859\pm 0.098\;\left(0.840\pm 0.089\right)}{0.720,0.793,0.905,1.090}\\ p1=0.12,\;p2=0.36 \end{array}$
	$L_{rem,Certyd}$	$\begin{array}{c} \frac{8.0\pm 1.3\;\left(8.0\pm 1.3\right)}{5.9,7.2,8.8,10.5}\\ p1=0.94,\;p2=0.93 \end{array}$	$\begin{array}{c} \frac{8.7\pm 1.3\;\left(9.0\pm 1.6\right)}{6.7,7.3,9.5,10.6}\\ p1=0.02 \end{array}$	$\begin{array}{c} \frac{0.817\pm 0.112\;\left(0.795\pm 0.082\right)}{0.670,0.743,0.865,1.070}\\ p1=0.03 \end{array}$
Non-veg. period	$L_{in}$	$\begin{array}{c} \frac{11.37\pm 1.27\;\left(10.97\pm 0.85\right)}{9.70,10.52,11.83,14.15}\\ p1<0.01 \end{array}$	$\begin{array}{c} \frac{9.37\pm 0.81\;\left(9.00\pm 0.58\right)}{8.50,8.83,9.65,11.65}\\ p1<0.01 \end{array}$	$\begin{array}{c} \frac{1.42\pm 0.28\;\left(1.41\pm 0.42\right)}{1.03,1.20,1.69,2.05}\\ p1=0.64,\;p2=0.16 \end{array}$
	$L_{rem,\;Gravel}$	$\begin{array}{c} \frac{6.31\pm 1.20\;\left(5.94\pm 0.95\right)}{4.97,5.51,6.91,9.11}\\ p1=0.01 \end{array}$	$\begin{array}{c} \frac{6.4\pm 1.1\;\left(6.3\pm 1.2\right)}{4.8,5.4,7.0,9.0}\\ p1=0.26,\;p2=0.31 \end{array}$	$\begin{array}{c} \frac{0.80\pm 0.21\;\left(0.80\pm 0.28\right)}{0.44,0.62,0.99,1.25}\\ p1=1.00,\;p2=0.65 \end{array}$
	$L_{rem,Certyd}$	$\begin{array}{c} \frac{6.46\pm 1.15\;\left(6.12\pm 0.76\right)}{5.27,5.67,7.08,9.16}\\ p1<0.01 \end{array}$	$\begin{array}{c} \frac{7.2\pm 1.1\;\left(7.1\pm 1.2\right)}{6.0,6.2,7.9,9.8}\\ p1=0.29,\;p2=0.09 \end{array}$	$\begin{array}{c} \frac{0.77\pm 0.18\;\left(0.76\pm 0.24\right)}{0.54,0.60,0.92,1.19}\\ p1=0.07,\;p2=0.16 \end{array}$

**Table 4:** Statistical comparison of treatment efficiencies.

	Period	Normality Test		Paired Test			Δ*η*
		W Statistics	*p*-Value	Version	Statistics	*p*-Value	
$\eta_{BOD_{5}}$	Non-veg	0.96281	0.4731	T	t = 10.362	$2.4\times 10^{-10}$	0.040
	Veg	0.91165	0.0920	T	t = 17.178	$3.5\times 10^{-12}$	0.027
$\eta_{COD}$	Non-veg	0.96815	0.5986	T	t = 7.468	$1.0\times 10^{-7}$	0.033
	Veg	0.95179	0.4538	T	t = 14.304	$6.6\times 10^{-11}$	0.029
$\eta_{TOC}$	Non-veg	0.91898	0.0486	Wilcoxon	V = 325	$1.3\times 10^{-5}$	0.076
	Veg	0.95450	0.5000	T	t = 9.7821	$2.1\times 10^{-8}$	0.042
$\eta_{N-NH_{4}}$	Non-veg	0.96365	0.4918	T	t = 14.618	$1.9\times 10^{-13}$	0.090
	Veg	0.84572	0.0073	Wilcoxon	V = 171	$7.6\times 10^{-6}$	0.016
$\eta_{TN}$	Non-veg	0.95978	0.4102	T	t = 2.1204	0.04451	0.014
	Veg	0.95945	0.5909	T	t = 4.347	$4.4\times 10^{-4}$	0.028
$\eta_{TP}$	Non-veg	0.94714	0.2160	T	t = −2.4828	0.02042	−0.021
	Veg	0.86944	0.0174	Wilcoxon	V = 11	$4.2\times 10^{-4}$	−0.031
$\eta_{SS}$	Non-veg	0.94302	0.5566	T	t = −3.845	0.003238	−0.040
	Veg	0.85908	0.0476	Wilcoxon	V = 0	$4.9\times 10^{-4}$	−0.082

**Table 5:** BOD_5_:COD and BOD_5_:TN ratios for the raw sewage and the treated sewage.

	BOD_5_:COD	BOD_5_:TN
In	$\begin{array}{c} \frac{0.555\pm 0.020\;\left(0.556\pm 0.021\right)}{0.514,0.541,0.568,0.597}\\ p1=0.72,\;p2=0.84 \end{array}$	$\begin{array}{c} \frac{3.57\pm 0.25\;\left(3.54\pm 0.21\right)}{2.96,3.44,3.69,4.10}\\ p1=0.89,\;p2=0.84 \end{array}$
Out Gravel	$\begin{array}{c} \frac{0.361\pm 0.061\;\left(0.356\pm 0.080\right)}{0.240,0.313,0.416,0.517}\\ p1=0.60,\;p2=0.39 \end{array}$	$\begin{array}{c} \frac{1.31\pm 0.39\;\left(1.21\pm 0.42\right)}{0.77,0.96,1.63,2.04}\\ p1=0.22,\;p2<0.01 \end{array}$
Out Certyd	$\begin{array}{c} \frac{0.320\pm 0.066\;\left(0.300\pm 0.069\right)}{0.229,0.266,0.382,0.449}\\ p1=0.04 \end{array}$	$\begin{array}{c} \frac{1.07\pm 0.39\;\left(0.95\pm 0.38\right)}{0.53,0.77,1.34,1.95}\\ p1=0.02 \end{array}$

**Table 6:** Dissolved oxygen concentration, pH, conductivity, and alkalinity of the raw sewage and treated sewage.

	DO mg·dm^−3^	pH	Conductivity $\mu S\cdot cm^{-1}$	Alkalinity $\mathrm{mval}\cdot dm^{-3}$
In	$\begin{array}{c} \frac{0.47\pm 0.24\;\left(0.45\pm 0.25\right)}{0.19,\;0.28,\;0.61,\;1.20}\\ p1=0.19,\;p2<0.01 \end{array}$	6.6,7.0,7.1,7.2,7.3	$\begin{array}{c} \frac{1490\pm 66\;\left(1480\pm 59\right)}{1320,1440,1518,1670}\\ p1<0.01 \end{array}$	$\begin{array}{c} \frac{13.6\pm 2.6\;\left(14.0\pm 3.0\right)}{7.9,12.0,16.0,16.9}\\ p1=0.19,\;p2=0.11 \end{array}$
Out Gravel	$\begin{array}{c} \frac{1.91\pm 0.56\;\left(1.80\pm 0.48\right)}{0.96,1.55,2.19,2.90}\\ p1=0.05,\;p2=0.37 \end{array}$	7.0,7.1,7.2,7.3,7.4	$\begin{array}{c} \frac{1331\pm 96\;\left(1327\pm 104\right)}{1196,1275,1390,1494}\\ p1=0.88,\;p2=0.81 \end{array}$	$\begin{array}{c} \frac{6.1\pm 1.6\;\left(6.6\pm 1.8\right)}{3.8,4.3,7.2,8.0}\\ p1=0.14,\;p2=0.04 \end{array}$
Out Certyd	$\begin{array}{c} \frac{2.43\pm 0.60\;\left(2.20\pm 0.65\right)}{1.62,1.90,3.00,3.60}\\ p1=0.57,\;p2=0.23 \end{array}$	7.1,7.2,7.3,7.3,7.4	$\begin{array}{c} \frac{1299\pm 51\;\left(1300\pm 44\right)}{1230,1260,1320,1423}\\ p1=0.12,\;p2=0.18 \end{array}$	$\begin{array}{c} \frac{7.38\pm 1.07\;\left(8.00\pm 0.74\right)}{5.00,6.85,8.03,9.00}\\ p1=0.05 \end{array}$

## Data Availability

Data are contained within the article.

## Abbreviations

CW—Constructed Wetlands, VF—Vertical Flow, CHP—Combined Heat and Power, SS-VF—Subsurface Vertical Flow, BOD_5_—Biological Oxygen Demand, COD—Chemical Oxygen Demand, TOC—Total Carbon, N-NH_4_—Ammonia Nitrogen, TN—Total Nitrogen, TP—Total Phosphorus, SSs—Suspended Solids, LECA—Light Expanded Clay Aggregate, LWA—Lightweight Aggregate.
